# Kidney and gallbladder stones and the risk of prostate cancer: Results from the EPICAP study

**DOI:** 10.1371/journal.pone.0317760

**Published:** 2025-01-17

**Authors:** Melissa Sawaya, Emilie Cordina-Duverger, Pierre-Jean Lamy, Brigitte Trétarre, Florence Menegaux

**Affiliations:** 1 UVSQ, Inserm, Gustave Roussy, CESP, Université Paris-Saclay, Villejuif, France; 2 Service de Recherche Clinique, Clinique Beau Soleil, Montpellier, France; 3 Service Urologie, Clinique Beau Soleil, Montpellier, France; 4 Registre des Tumeurs de l’Hérault, EA 2415, ICM, Montpellier, France; 5 Center for Epidemiology and Research in Population Health (CERPOP), Toulouse, France; Khyber Medical University, PAKISTAN

## Abstract

**Background:**

Prostate cancer remains the most frequent cancer among men, representing a significant health burden. Despite its high morbidity and mortality rates, the etiology of prostate cancer remains relatively unknown, with only non-modifiable established risk factors. Chronic inflammation has emerged as a potential factor in prostate carcinogenesis. We investigated the role of kidney and gallbladder stones and the risk of prostate cancer.

**Methods:**

We used data from EPICAP, a population-based case-control study. A total of 819 diagnosed prostate cancer cases and 879 controls were face-to-face interviewed using a standardized questionnaire that collected information on personal medical history, including history of kidney and gallbladder stones. Odds Ratios (OR) and their 95% confidence interval (CI) were estimated using multivariate unconditional logistic regression.

**Results:**

Our study revealed intriguing patterns regarding kidney and gallbladder stones in relation to prostate cancer risk. The analysis indicated significant potential associations between kidney stones and the risk of prostate cancer (OR: 1.46 95% CI: 1.13–1.90), particularly in men with a history of kidney infection. Additionally, our data suggested a possible relationship between gallbladder stones and prostate cancer when considering triglyceride (OR: 2.27, 95% CI: 0.99–5.28), although further research is needed for a conclusive understanding.

**Conclusions:**

Our results suggest an association between calculi and the risk of prostate cancer. Findings from this study underscore the need for a more comprehensive investigation to understand the role of chronic inflammation or metabolism and delineate the mechanisms underlying these potential associations in order to guide the development of targeted preventive strategies for aggressive prostate cancer.

## Introduction

Prostate cancer remains the most frequent cancer among men in the Western world, representing a significant health burden. In 2022, approximately one million cases of prostate cancer were reported worldwide, resulting in nearly 400,000 deaths [1, 2]. In France, prostate cancer is classified as the 2^nd^ highest cause of cancer-related deaths, contributing to more than 57,000 diagnosed new cases and 9,000 deaths during the same year [1–3].

While prostate cancer exhibits elevated morbidity and mortality rates, our understanding of its etiology remains notably limited. The main well-established non-modifiable risk factors are ethnicity, advanced age, and family history of prostate cancer [4]. Research on migrant populations has shown that Asian men residing in the United States have a significantly higher incidence of prostate cancer than those residing in their home countries, implying that lifestyle and environmental factors may play a role [5]. Various studies in epidemiology have proposed that prostate inflammation could be linked to the development of prostate cancer [6, 7]. Inflammatory cells found near areas of proliferative inflammatory atrophy and prostatic intraepithelial neoplasia—both considered precancerous lesions—further support the hypothesis that chronic inflammation may contribute to prostate cancer development [2, 6, 8].

Urinary stone disease involves the formation of mineral and salt deposits within the urinary tract, including stones that can occur in the kidney, ureter, and urinary bladder [9]. It is a common urological condition in which many experience recurrent episodes [10]. Kidney stones represent a prevalent urological condition affecting 5 to 10% of the population, influenced by factors such as dietary habits, metabolic conditions, urinary infections, environmental factors, and genetic predisposition [11]. Although earlier population-based studies have shown that an increased risk of urinary tract cancers is associated with having a history of urinary calculi, our understanding of the potential connection between urinary stones and systemic cancers remains relatively unclear [12]. Limited research has investigated the possible role of kidney stones in the occurrence of prostate cancer, and the findings have been inconclusive. For instance, a study conducted in Italy indicated an increased risk of prostate cancer with kidney stones, even though not significant [13], while two Taiwanese studies reported a significantly high-risk [12, 14].

Gallbladder stones are hardened deposits that can vary in size and composition, usually consisting of cholesterol, bilirubin, calcium salts, and other substances [15, 16]. According to recent estimates, approximately 10 to 15% of the adult population is affected by gallbladder stones [15]. A recent meta-analysis examined a total of 7 studies investigating the relationship between gallbladder stones and prostate cancer, which observed significant associations reinforcing the hypothesis of a possible link with prostate cancer, yet none of these studies addressed the aggressiveness of cancer [17]. One possible explanation is that chronic inflammation associated with gallbladder stones may release inflammatory mediators that, when systemically distributed, could influence the prostate microenvironment, potentially promoting favorable conditions for prostate cancer initiation [18]. Another possible explanation is that elevated cholesterol levels in individuals with gallbladder stones can accumulate not only in the gallbladder but also systemically, potentially infiltrating the prostate tissues which may trigger oncogenic processes [19].

In light of this, we aim at examining the different associations between either kidney or gallbladder stones and the risk of prostate cancer, while focusing on the aggressive types using data from the EPICAP study.

## Methods

### Study population

EPICAP (EPIdemiological study of Prostate CAncer) is a population-based case-control study conducted in the Hérault department located in the south of France between 2012 and 2013 to investigate the role of environmental and genetic factors in the occurrence of prostate cancer. Details of the EPICAP objectives and study design have been previously described [20].

The eligible cases were men diagnosed with incident prostate cancer between 2012 and 2013, were under 75 years old, and were residents of the Hérault department at the time of diagnosis. All cases included in the study were confirmed through histological examination. Clinical research nurses identified cases through all public and private cancer care centers within the department. Controls were randomly selected from the general population, were not diagnosed with prostate cancer, and residing in the same department at the time of inclusion. To ensure frequency-matching, age quotas were established in advance, yielding a control group that is comparable to the case group in terms of age (5-year age group). Additionally, socioeconomic status (SES) quotas were predefined to create a control group comparable to the general male population of the same age in the Hérault department.

In total, EPICAP had 1,098 incident prostate cancer cases and 1,109 population-based controls that were eligible, of which 819 cases and 879 controls were included, with a participation rate of 75% and 79%, respectively.

#### Ethical statement

All participants provided informed written consent. The EPICAP study was approved by the review board of the French Institute of Health and Medical Research (INSERM, n°01–040, November 2010) and authorized by the French data protection authority (CNIL n°910485, April 2011).

#### Data collection

Cases and controls were interviewed face-to-face by a well-trained clinical research nurse using a standardized computer-assisted questionnaire. Information on sociodemographic and lifestyle characteristics (diet, tobacco, alcohol consumption, and physical activity), occupational and residential history, family history of prostate cancer, and anthropomorphic measurements were collected.

Regarding personal medical history, self-reporting of a personal history of kidney and gallbladder stones as well as other conditions such as urinary infections (prostatitis, pyelonephritis) [17] and dyslipidemia (hypercholesterolemia, hypertriglyceridemia) [18] were also collected.

Clinical medical information for each case, such as Gleason scores and Prostate Specific Antigen (PSA), were extracted from the patient’s medical records at the time of diagnosis and validated by the Hérault cancer registry at posteriori.

#### Statistical analysis

All analyses were performed using the statistical analysis software SAS (9.4 version).

The personal history of kidney and gallbladder stones was defined individually based on whether the individuals had ever experienced any of these conditions before the reference date (the date of diagnosis for the cases and the date of the interview for the controls). A global variable defined by “Calculi” was done by grouping together the occurrence of kidney and gallbladder stones, aiming to provide a comprehensive view of their association with the risk of prostate cancer.

To gain a better understanding of the underlying mechanisms of chronic inflammation, we explored the various associations of kidney and gallbladder stones with prostate cancer by analyzing the combination of these factors and applying stratification.

Odds ratios (OR) and their 95% confidence intervals (95% CI) were computed using unconditional logistic regression to investigate the association between kidney and gallbladder stones and the risk of prostate cancer. The analysis was systematically adjusted to the established risk factors such as age, ethnicity (Caucasians, others), and family history of prostate cancer in first-degree relatives, as well as potential confounding factors such as education, physical activity, non-steroidal anti-inflammatory drugs (NSAIDs) and waist circumference (WC).

Analysis was also performed to assess the relationship based on the aggressiveness of prostate cancer using the Gleason score at the time of diagnosis [19]. For non-aggressive or low-grade cancer, the Gleason score was ≤ 7 [including 3 + 4], which is considered a low or intermediate score; for aggressive or high-grade cancer, the Gleason score was ≥ 7 [including 4 + 3], which is regarded as a high score.

## Results

Table 1 shows the characteristics of controls and prostate cancer cases within the EPICAP. Within the cases, 22.7% of men were identified as having aggressive or high-grade cancer. The study population was primarily made up of individuals of Caucasian descent, accounting for approximately 97.0% of both cases and controls. Potential confounding factors such as education, Body Mass Index, physical activity, waist circumference, and hypercholesterolemia were quite comparable between cases and controls. Non-steroidal anti-inflammatory drugs and hypertriglyceridemia were more frequent among controls than in cases. As anticipated, a family history of prostate cancer in first-degree relatives was significantly higher in cases (22.2%) compared to controls (8.8%).

**Table 1:** Study population characteristics of EPICAP.

	Cases n = 819 (%)	Controls n = 879 (%)	P-value
**Gleason Score**			
≤ 7 (3+4)	623 (77.3)	-	
≥7 (4+3)	183 (22.7)	-	
**Age (Years)**			*0*.*144*
< 55	48 (5.9)	59 (6.7)	
[55–60]	99 (12.1)	99 (11.2)	
[60–65]	217(26.5)	201 (22.9)	
[65–70]	274 (33.5)	285 (32.4)	
≥70	181 (22.1)	235 (26.7)	
**Ethnic Origin**			*0*.*396*
Caucasian	795 (97.1)	859 (97.7)	
Other	24 (2.9)	20 (2.3)	
**Family History of Prostate Cancer**			*<0*.*001*
No	633 (77.8)	799 (91.2)	
Yes	181 (22.2)	77 (8.8)	
**Education**			*0*.*609*
Primary	179 (21.9)	193 (22.0)	
Secondary	380 (46.4)	425 (48.4)	
University	260 (31.8)	260 (29.6)	
**Body Mass Index (kg/m** ^**2**^ **)**			*0*.*534*
<25—Normal	231 (28.5)	248 (29.1)	
[25–30]—Overweight	399 (49.1)	397 (46.6)	
≥30—Obese	182 (22.4)	207 (24.3)	
**Waist Circumference (cm)**			*0*.*086*
≤94	209 (25.9)	254 (29.6)	
>94	599 (74.1)	603 (70.4)	
**Smoking Status**			*0*.*288*
Never	240 (29.3)	246 (28)	
Former	455 (55.6)	476 (54.2)	
Current	123 (15.0)	157 (17.9)	
**Alcohol Consumption**			*0*.*591*
No	72 (8.8)	84 (9.6)	
Yes	746 (91.2)	795 (90.4)	
**Physical Activity**			*0*.*109*
No	191 (23.4)	177 (20.1)	
Yes	627 (76.7)	702 (79.9)	
**Nonsteroidal anti-inflammatory drugs**			*0*.*043*
No	596 (73.0)	593 (68.6)	
Yes	220 (27.0)	272 (31.5)	
**Hypertriglyceridemia**			0.037
No	668 (81.6)	681 (77.5)	
Yes	151 (18.4)	198 (22.5)	
**Hypercholesterolemia**			0.263
No	474 (57.9)	496 (56.4)	
Yes	343 (41.9)	376 (42.8)	

Table 2 presents the relationships between kidney stones, gallbladder stones, and the risk of prostate cancer in all its grades. Among all prostate cancer cases, a prior history of kidney stones significantly increased the risk of prostate cancer (OR: 1.46 95% CI: 1.13–1.90). This risk remained significant when considering low-grade cancer (OR: 1.49, 95% CI: 1.13–1.98), while the significant association was no longer observed when considering high-grade cancer (OR: 1.16, 95% CI: 0.75–1.80). A prior history of gallbladder stones did not show a significant association with the risk of prostate cancer (OR: 1.19, 95% CI: 0.83–1.70), including both low-grade (OR: 1.14, 95% CI: 0.77–1.69) and high-grade (OR: 1.43, CI: 0.83–2.45) cancers. For individuals with a history of calculi overall, the risk of prostate cancer was significant across all grades (OR: 1.39, 95% CI: 1.10–1.75) and low-grade (OR: 1.42, 95% CI: 1.11–1.82).

**Table 2:** Association between kidney stones, gallbladder stones, calculi, and the risk of prostate cancer.

Variables	Control	Cases					
		ALL		Low-Grade**		High-Grade***	
	N = 879	N = 819	OR* (95%CI)	N = 623	OR* (95%CI)	N = 183	OR* (95%CI)
**Kidney Stones**							
No	732 (84.1)	639 (78.5)	*1*.*00 reference*	487 (78.6)	*1*.*00 reference*	146 (80.2)	*1*.*00 reference*
Yes	138 (15.9)	175 (21.5)	**1.46 (1.13–1.90)**	133 (21.5)	**1.49 (1.13–1.98)**	36 (19.8)	1.16 (0.75–1.80)
**Gallbladder stones**							
No	802 (92.3)	743 (91.3)	*1*.*00 reference*	569 (91.9)	*1*.*00 reference*	161 (88.5)	*1*.*00 reference*
Yes	67 (7.7)	71 (8.7)	1.19 (0.83–1.70)	50 (8.1)	1.14 (0.77–1.69)	21 (11.5)	1.43 (0.83–2.45)
**Calculi** ^a^							
No	673 (77.7)	583 (71.9)	*1*.*00 reference*	44 (72.0)	*1*.*00 reference*	133 (73.1)	*1*.*00 reference*
Yes	193 (22.3)	228 (28.1)	**1.39 (1.10–1.75)**	173 (28.0)	**1.42 (1.11–1.82)**	49 (26.9)	1.17 (0.80–1.72)

*Adjusted on Age, Ethnicity, and family history of prostate cancer, Physical Activity, NSAIDs, Waist circumference, Education, Hypertriglyceridemia

** Gleason ≤ 7 (3+4)

*** Gleason ≥ 7 (4+3)

^a^ Calculi: Kidney Stones, Gallbladder Stones

Table 3 shows the associations between combined variables examining simultaneously kidney stones, urinary infections (prostatitis and pyelonephritis), and prostate cancer risk. When combined with a personal history of prostatitis and kidney stones, the association was no longer observed with prostate cancer (OR: 1.14, 95% CI: 0.57–2.29), while associations between kidney stones and prostate cancer were increased in the absence of a personal history of prostatitis (OR: 1.59, 95% CI: 1.21–2.10). However, even though based on small numbers, when combined with a personal history of pyelonephritis, kidney stones were more strongly associated with prostate cancer (OR: 5.43, 95% CI: 1.16–25.4) than in the absence of a personal history of pyelonephritis (OR: 1.41, 95% CI: 1.08–1.83). Interestingly, associations with the absence of a personal history of pyelonephritis were higher specifically for low-grade prostate cancer (OR: 1.47, 95% CI: 1.10–1.95), while the simultaneous presence of a personal history of pyelonephritis was higher when taking into account high-grade prostate cancer only (OR: 10.9, 95% CI: 1.94–61.3) despite being based on a small sample size.

**Table 3:** Combined variables with kidney stones and the risk of prostate cancer.

Variables	Control	Cases					
		ALL		Low-Grade**		High-Grade***	
	N = 879	N = 819	OR* (95%CI)	N = 623	OR* (95%CI)	N = 183	OR* (95%CI)
**Kidney Stones (KS) and Prostatitis**							
No KS / No Prostatitis	687 (79.0)	572 (70.3)	*1*.*00 reference*	437 (70.5)	*1*.*00 reference*	129 (70.9)	*1*.*00 reference*
No KS / Prostatitis +	45 (5.2)	67 (8.2)	**1.74 (1.16–2.61)**	50 (8.1)	**1.66 (1.07–2.57)**	17 (9.3)	**2.08 (1.13–3.81)**
KS + / No Prostatitis	120 (13.8)	158 (19.4)	**1.59 (1.21–2.10)**	118 (19.0)	**1.59 (1.18–2.15)**	34 (18.7)	1.33 (0.84–2.10)
KS + / Prostatitis +	18 (2.1)	17 (2.1)	1.14 (0.57–2.29)	15 (2.4)	1.33 (0.64–2.75)	2 (1.1)	0.57 (0.13–2.54)
**Kidney Stones (KS) and Pyelonephritis (Pyelo)**							
No KS / No Pyelo	717 (83.1)	623 (76.7)	*1*.*00 reference*	474 (76.7)	*1*.*00 reference*	143 (78.6)	*1*.*00 reference*
No KS / Pyelo +	9 (1.04)	15 (1.9)	1.86 (0.79–4.38)	12 (1.9)	2.00 (0.81–4.91)	3 (1.7)	1.53 (0.40–5.86)
KS + / No Pyelo	135 (15.6)	164 (20.2)	**1.41 (1.08–1.83)**	127 (20.6)	**1.47 (1.10–1.95)**	31 (17.0)	1.00 (0.63–1.58)
KS + / Pyelo +	2 (0.2)	10 (1.2)	**5.43 (1.16–25.4)**	5 (0.8)	3.69 (0.67–20.2)	5 (2.8)	**10.90 (1.94–61.3)**

*Adjusted on Age, Ethnicity, and family history of prostate cancer, Physical Activity, NSAIDs, Education, waist circumference

** Gleason ≤ 7 (3+4)

*** Gleason ≥ 7 (4+3)

In Table 4, the Odds Ratios (ORs) present the association between kidney and gallbladder stones and the risk of prostate cancer, considering stratification by triglyceride status. For individuals with normal triglyceride status, the presence of gallbladder stones did not significantly alter the risk of prostate cancer across all grades. Specifically, in cases of both low-grade (OR: 0.95, 95% CI: 0.62–1.51) and high-grade (OR: 1.21, 95% CI: 0.66–2.25) cancers, the odds ratios indicated no significant association with gallbladder stones. However, in individuals with hypertriglyceridemia, the presence of gallbladder stones significantly increased the risk of prostate cancer. While it was borderline significant among all cases (OR: 2.27, 95%CI: 0.99–5.28) and not significant for low-grade (OR: 2.07, 95% CI: 0.83–5.17), the risk was notably higher for high-grade cancer (OR: 3.58, 95% CI: 1.05–12.22). Regarding kidney stones, specifically for all cases (OR: 1.69, 95%CI: 1.26–2.20) and low-grade (OR: 1.75, 95%CI: 1.27–2.40), the association was significant among individuals with normal triglyceride. The association was lost among individuals with hypertriglyceridemia in all cases (OR: 0.84, 95%CI: 0.47–1.50) and low-grade prostate cancer (OR:0.78, 95%CI: 0.41–1.49).

**Table 4:** Association between kidney and gallbladder stones and the risk of prostate cancer when stratifying on triglyceride status.

		*Controls*	*Cases*					
			ALL		Low grade**		High-grade***	
		**N = 673**	**N = 666**	OR* **(95% CI)**	**N = 507**	OR* **(95% CI)**	**N = 147**	OR* **(95% CI)**
**Normal Triglyceride Status, N = 1338 g**	**Gallbladder stones**							
	No	617 (91.7)	612 (92.0)	1.00 Reference	468 (92.5)	1.00 Reference	132 (89.8)	1.00 Reference
	Yes	56 (8.3)	53 (8.0)	0.99 (0.66–1.48)	38 (7.5)	0.95 (0.62–1.51)	15 (10.2)	1.21 (0.66–2.25)
	**Kidney Stones**							
	No	573 (85.3)	517 (77.6)	1.00 Reference	392 (77.3)	1.00 Reference	119 (81.0)	1.00 Reference
	Yes	99 (14.7)	149 (22.4)	**1.69 (1.26–2.20)**	115 (22.7)	**1.75 (1.27–2.40)**	28 (19.0)	1.21 (0.74–1.99)
		**N = 198**	**N = 149**	OR* **(95% CI)**	**N = 113**	OR* **(95% CI)**	**N = 35**	OR* **(95% CI)**
**Hypertriglyceridemia Status, N = 346**	**Gallbladder stones**							
	No	185 (94.4)	131 (87.9)	1.00 Reference	101 (89.4)	1.00 Reference	29 (82.3)	1.00 Reference
	Yes	11 (5.6)	18 (12.1)	**2.27 (0.99–5.28)**	12 (10.6)	2.07 (0.83–5.17)	6 (17.1)	**3.58 (1.05–12.22)**
	**Kidney Stones**							
	No	159 (80.3)	122 (82.4)	1.00 Reference	95 (84.1)	1.00 Reference	27 (77.1)	1.00 Reference
	Yes	39 (19.7)	26 (17.6)	0.84 (0.47–1.50)	18 (15.9)	0.78 (0.41–1.49)	8 (22.9)	1.19 (0.46–3.08)

*Adjusted on Age, Ethnicity, and family history of prostate cancer, Physical Activity, NSAIDs, Education, waist circumference

** Gleason ≤ 7 (3+4)

*** Gleason ≥ 7 (4+3)

## Discussion

When considering triglyceride status, the presence of gallbladder stones significantly increased the risk of prostate cancer in individuals with hypertriglyceridemia, especially in high-grade cancers. The potential connection between elevated triglyceride status, gallbladder stones, and prostate cancer could be multifaceted. Triglycerides, a key component of lipid metabolism, often exhibit elevation in individuals with metabolic syndrome, a well-known risk factor for both gallbladder stones and prostate cancer [21, 22]. This association may indicate a shared metabolic pathway underlying the two conditions. Elevated triglyceride status is often associated with gallbladder stone formation, which may influence the pro-inflammatory and pro-oxidative conditions observed in both conditions, thereby promoting an environment favorable to prostate carcinogenesis [23–25].

Our results showed an association between a personal history of kidney stones and an increased risk of prostate cancer, strengthened by the presence of a personal history of pyelonephritis but not prostatitis. Few studies have explored the relationship between a history of kidney stones and the risk of prostate cancer. Our results align with two previous studies that yielded a potential link between kidney stones and prostate cancer risk [12, 14]. While our findings demonstrated a significant association between a history of kidney stones and an increased risk of prostate cancer, not all studies have yielded similar results. For instance, a hospital-based case-control study in Italy found no significant association between urinary tract stones and prostate cancer risk [13].

Similarly, a cohort study conducted in Sweden also indicated that a history of kidney stones was not significantly linked to prostate cancer risk [26]. One plausible explanation for this connection could be the shared underlying metabolic and lifestyle factors predisposing individuals to both conditions [27–29].

For instance, dietary factors could have influenced alterations in the urinary composition favoring kidney stone formation, which in turn could have triggered hormonal shifts that advanced the development of prostate cancer [14]. Another potential explanation for the observed association could involve chronic inflammation as a common pathway. Kidney stones may cause mechanical irritation or obstruct the urinary tract, leading to urine retention and stasis. This can create a favorable environment for bacterial colonization, further exacerbating inflammation [30, 31]. Chronic inflammation, in turn, has been associated with DNA damage, cellular proliferation, and the progression of tumorigenesis [31–33]. Additionally, the presence of pyelonephritis, a bacterial infection of the kidney, may further amplify this inflammatory response [34]. Pyelonephritis can cause significant and recurrent inflammation in the urinary tract, which may synergize with the effects of kidney stones to create a prolonged pro-inflammatory environment [35]. This persistent inflammation could promote oxidative stress, the release of inflammatory mediators, and DNA damage, facilitating cancer development [36, 37]. Further, hormonal imbalances linked to altered calcium metabolism, including elevated parathyroid hormone or vitamin D dysregulation, may influence prostate cell proliferation. These hormonal shifts could act as mediators between kidney stone formation and prostate cancer risk [26, 32].

Contrarily, a personal history of gallbladder stones did not demonstrate a significant link with the overall risk of prostate cancer, including both low-grade and high-grade cancers, consistent with previous studies [17, 38]. However, a recent meta-analysis showed a pooled relative risk (RR) of 1.35 (95% CI: 1.17–1.56), which did not align with our study findings [17]. While the overall association between a history of gallstones and prostate cancer was not significant, the stratified analysis by triglyceride status revealed another perspective.

Our results are derived from a large and well-designed population-based case-control study aimed at investigating environmental as well as genetic factors and the risk of prostate cancer, while specifically targeting its most aggressive types. The identification of cases encompassed all private and public cancer centers in the Hérault department. In 2011, the Hérault cancer registry recorded 770 new cases of prostate cancer in men, with 75% being under 75 years old. During the study period, assuming a similar case incidence in 2012–2013, an estimated 1150 new cases were expected.

The exhaustive recruitment of cases resulted in 1,098 eligible cases throughout the study, minimizing the risk of selection bias. To match the age distribution of the cases, controls were chosen randomly from the general population of the Hérault department, following 5-year age quotas. Additionally, the distribution of the socioeconomic status of the control group was aligned with that of the Hérault department, ensuring it was comparable to the general population to minimize selection bias as well. Following the selection process, comparing the socioeconomic status distribution of the control group with that of the general male population in the Hérault department revealed no major difference, indicating the absence of major selection bias by socioeconomic status in our controls.

To address potential detection bias, sensitivity analyses were conducted, particularly because our findings showed a positive association between kidney stones and prostate cancer limited to low-grade cases, with no significant associations observed for high-grade cases. Detection bias is a known challenge in prostate cancer research, as increased medical surveillance or diagnostic investigations, such as PSA testing, following a history of kidney stones, could lead to the incidental detection of asymptomatic, low-grade tumors. This heightened surveillance may overestimate the association between kidney stones and prostate cancer risk by disproportionately identifying low-grade cases that might otherwise have gone undetected. When we restriceted our analyses to controls who had undergone PSA testing within the last two years, or controls with frequent PSA testing, we did not observed differences in our results minimizing a potential detection bias in our results.

Considering the risk of recall bias in our study is crucial, especially since the data collection method relies on self-reporting, which may introduce classification bias, either differential or non-differential. To mitigate this bias, face-to-face interviews were conducted using a standardized questionnaire by a trained research clinical nurse, ensuring the process was identical for both cases and controls. However, in the EPICAP study, the prevalence regarding the control group was identified as 7.7% for gallbladder stones. Given the unavailability of precise data on gallbladder stone prevalence in France, we compared our findings with those from other relevant studies. In a previous study conducted with a similar demographic, the prevalence of gallbladder stones among controls was 6.2%, indicating a relatively similar prevalence [38]. This helps mitigate the potential impact of recall/classification bias in our findings. In addition, the prevalence of kidney stones among controls observed in our study was around 15.9%, which was also similar to the prevalence of kidneys at 13.6% in the population of France [39]; this also helps minimize the potential impact of recall/classification bias in our findings.

Our study has several limitations that should be considered when interpreting the results. First, we lacked detailed information on the characteristics of stones, such as their type and size, which could influence both the severity of symptoms and the likelihood of detection. We also faced limitations due to the lack of data on the timing of infections and the diagnosis of stones, which restricted our ability to differentiate between short-term and long-term effects on prostate cancer risk. This limitation may have left residual confounding factors unaccounted for. Another limitation was the reduced statistical power in some analyses, particularly those involving smaller sample sizes, such as stratified analyses and those focused on high-grade prostate cancer. This constraint may have limited our ability to detect certain associations, potentially underestimating the true relationships in specific subgroups. Finally, our findings remained consistent even after accounting for non-modifiable risk factors and potential confounding variables, including factors like NSAIDs, waist circumference, education level, and physical activity.

## Conclusion

In summary, our study showed significant associations between kidney stones and prostate cancer, particularly in men with a personal history of pyelonephritis and a kidney infection, reinforcing the possible role of chronic inflammation in prostate carcinogenesis. The nuanced analysis of gallbladder stones, particularly concerning elevated triglyceride status, highlighted the complex nature of these connections. These findings point to the broader implications for monitoring and prevention in specific populations, underscoring the necessity for further research to unravel the underlying mechanisms connecting kidney stones, gallbladder stones, and prostate cancer.

## Abbreviations

BMI: Body Mass Index
CI: Confidence Interval
CNIL: French data protection authority
EPICAP: EPIdemiology of Prostate CAncer
INSERM: French institute of health and medical research
NSAIDs: non-steroidal anti-inflammatory drugs
OR: Odds Ratio
PSA: Prostate Specific Antigen
SES: Socioeconomic Status
WC: waist circumference
